# Massive hemothorax following CT-guided lung biopsy: A rare iatrogenic complication managed conservatively

**DOI:** 10.1016/j.tcr.2026.101303

**Published:** 2026-02-12

**Authors:** Bahman Rasuli, Ali Forat Yazdi

**Affiliations:** a Department of Radiology, School of Medicine, Advanced Diagnostic and Interventional Radiology Research Center, Tehran University of Medical Sciences, Tehran, Iran; b Department of Radiology, School of Medicine, Advanced Diagnostic and Interventional Radiology Research Center, Iran University of Medical Sciences, Tehran, Iran

**Keywords:** Trauma, Hemothorax, CT-guided lung biopsy, Chest tube drainage, Iatrogenic complications

## Abstract

CT-guided transthoracic lung biopsy is an essential diagnostic technique for evaluating pulmonary lesions. Although rare, major complications such as hemothorax can be life-threatening. We report the case of a 35-year-old woman who developed a rapidly enlarging right hemothorax several hours after CT-guided core needle biopsy of a pleura-abutting right lower lobe mass. There was no arterial extravasation, and chest tube drainage yielded approximately 1500 mL of dark, clotted blood, suggesting a venous or tumoral source. The patient remained hemodynamically stable and achieved complete recovery with conservative management. This case highlights that even massive post-biopsy hemothorax can be successfully treated non-operatively in stable patients by adhering to trauma-based management principles.

## Introduction

CT-guided percutaneous lung biopsy is a minimally invasive and widely accepted diagnostic procedure for pulmonary lesions, providing high diagnostic accuracy with a low complication rate. Nevertheless, complications such as pneumothorax, pulmonary hemorrhage, and, rarely, hemothorax may occur. The reported incidence of hemothorax is less than 0.3% [1], [2].

Most hemothoraces following lung biopsy result from iatrogenic injury to intercostal or internal mammary arteries. In contrast, venous or tumoral bleeding particularly in pleura-adjacent lesions is uncommon and may present with delayed symptoms rather than immediate hemodynamic instability. Reported risk factors for bleeding include lower-lobe location, pleural contact, and use of large-bore needles [3], [4].

This report describes a delayed, massive hemothorax following CT-guided biopsy of a right lower-lobe mass. We detail the clinical presentation, imaging findings, and successful conservative management, with a discussion on potential bleeding mechanisms and their relation to trauma-based management principles [5].

## Case report

A 35-year-old woman presented with a slowly enlarging right lower-lobe mass, incidentally detected during evaluation for persistent cough and chest discomfort. She had no significant medical history, was not taking anticoagulants, and had normal coagulation parameters (PT, PTT, and INR within reference range). Physical examination and vital signs were unremarkable, and no signs of respiratory distress were present.

**Fig. 1:**
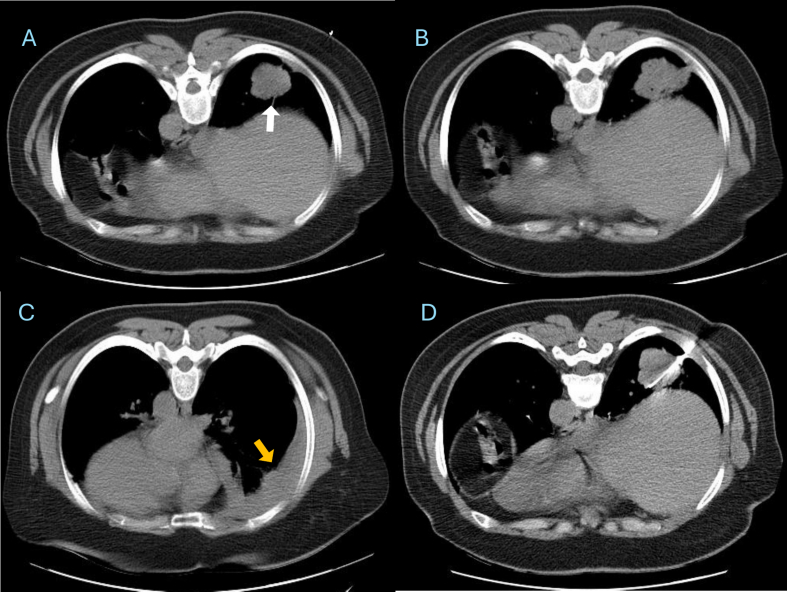
Axial CT images in the prone position showing a peripheral right lower-lobe lung mass (white arrow) immediately before and during CT-guided core needle biopsy. A minimal right pleural effusion is visible (yellow arrow). (For interpretation of the references to colour in this figure legend, the reader is referred to the web version of this article.)

Initial non-contrast chest CT showed a lobulated isodense mass in the right lower lobe basal segments with pleural contact. Minimal hypodense pleural fluid was also present (Fig. 1).

**Fig. 2:**
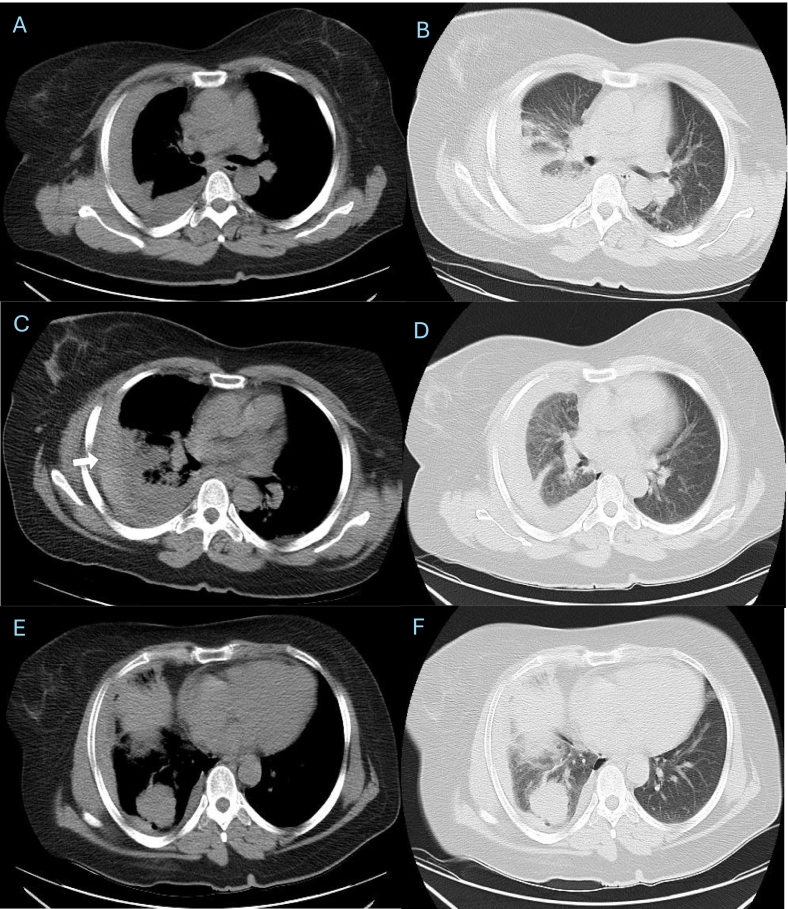
Axial CT images in the supine position obtained 30 min post-biopsy showing increased right pleural fluid. The newly developed high-density component indicates early hemorrhagic transformation (white arrow).

CT-guided core needle biopsy was performed using an 18-gauge coaxial cutting needle under local anesthesia with the patient in the prone position (Fig. 1). Two core samples were obtained without immediate complications. A post-procedure non-contrast CT obtained 30 min later showed a mild right-sided pleural effusion of mixed attenuation, suggesting early hemorrhage (Fig. 2). The patient remained stable and asymptomatic at that time.

**Fig. 3:**
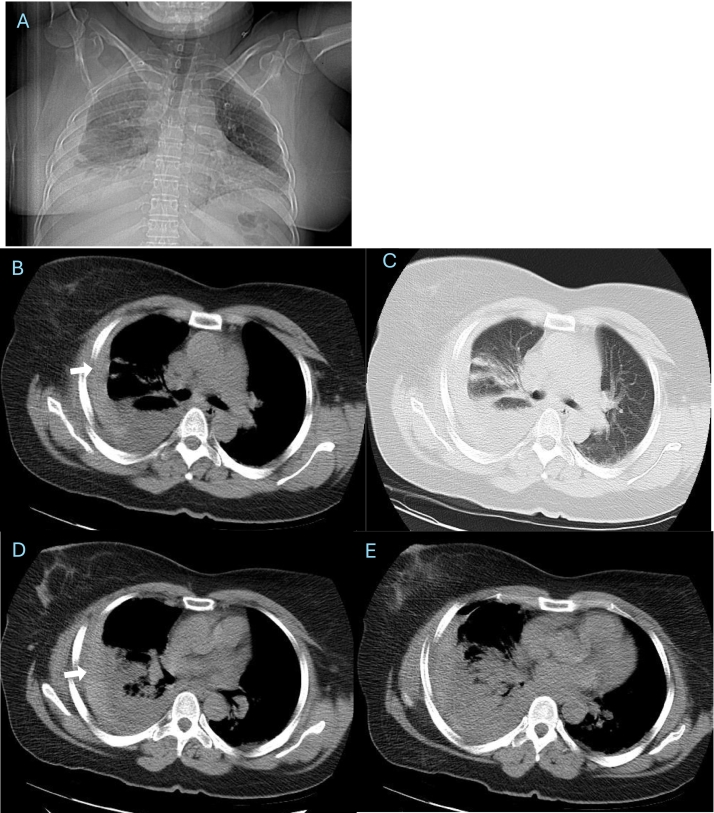
Scout and axial CT images in the supine position acquired 6 h after biopsy showing substantial enlargement of the right pleural effusion (white arrow) with compressive atelectasis of the lower lobe. The persistent high-density fluid supports ongoing hemothorax.

**Fig. 4:**
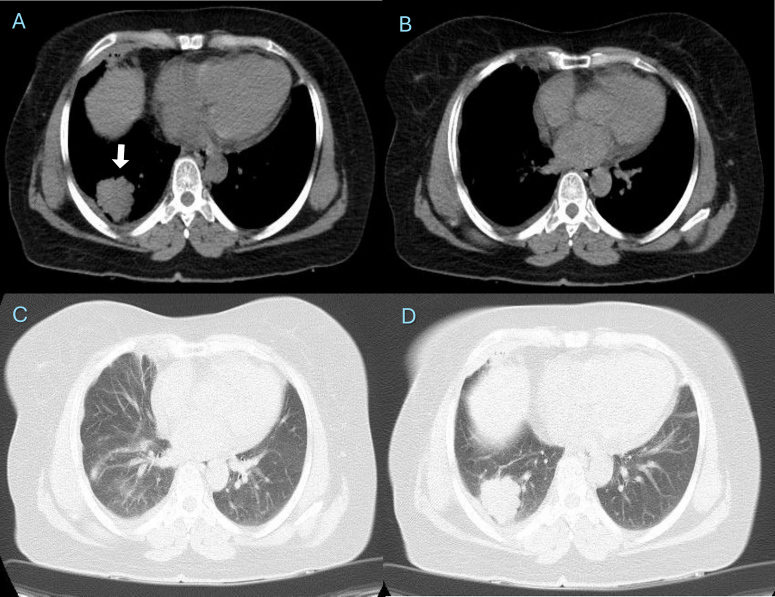
Axial CT images in the supine position performed 14 days post-biopsy showing near-complete resolution of the hemothorax and a stable right lower-lobe mass without recurrent fluid accumulation.

Approximately 6 h later, she developed worsening right-sided chest pain and dyspnea. A follow-up CT revealed a large right pleural effusion with homogeneous high attenuation consistent with hemothorax (Fig. 3). Her oxygen saturation decreased to 87%, and hemoglobin dropped from 13 to 9 g/dL. After surgical consultation, a chest tube was inserted, draining about 1500 mL of dark, partially clotted blood within 24 h. Although no contrast-enhanced CT was performed, the gradual accumulation and nature of the drained blood suggested a venous or tumoral source rather than arterial injury. The patient remained hemodynamically stable and was managed conservatively with oxygen therapy, intravenous fluids, and close monitoring. Chest tube output gradually decreased to <100 mL/day and was removed on day 3. Follow-up CT after 14 days demonstrated near-complete resolution of the hemothorax (Fig. 4).

Histopathologic analysis of the biopsy specimens revealed primary pulmonary adenocarcinoma. Microscopic evaluation showed tumor cell infiltration consistent with adenocarcinoma, with positive immunohistochemical staining for TTF-1 and Napsin A, confirming the diagnosis. The patient was subsequently referred to the radio-oncology service for further management.

## Discussion

Hemothorax following CT-guided lung biopsy is rare but potentially serious, with an incidence ranging from 0.05% to 0.3%. Most cases are due to injury of intercostal or internal thoracic arteries. In the absence of bright red, pulsatile drainage or active extravasation on imaging, venous or tumoral bleeding should be suspected, as in this case. Venous bleeding typically causes delayed accumulation due to low-pressure flow [6], [7].

Technical and anatomical risk factors include lower-lobe or pleura-adjacent lesions, use of larger needles, and traversing intercostal vessels. Other non-iatrogenic causes of hemothorax such as trauma, vascular malformations, neoplasms, and coagulopathies should be considered in the differential diagnosis [8], [9].

Management should follow trauma-based principles. Tube thoracostomy remains the mainstay for diagnosis and treatment. Surgical intervention or embolization is indicated for persistent bleeding (>200–300 mL/h for 3 h or >1500 mL initial drainage). Stable patients without arterial bleeding can often be managed conservatively. In this patient, conservative treatment led to full recovery, illustrating that even massive hemothorax can resolve without surgery [10], [11].

Performing a limited non-contrast CT scan shortly after biopsy can be valuable for detecting minor complications such as small pneumothoraces or limited hemothoraces before they progress. In our case, an early post-biopsy CT revealed initial hemorrhage, prompting closer observation. Over the following hours, gradual respiratory decline was noted, leading to timely chest tube placement. The character of the drained blood can help infer the bleeding source bright red, high-flow output suggests arterial injury, whereas dark, clotted blood with slower drainage points to venous or tumoral bleeding, as seen here. Conservative management with drainage, observation, and supportive care for 48–72 h may be sufficient in stable patients.

Although contrast-enhanced CT is generally recommended when active bleeding or arterial injury is suspected, a non-contrast CT was selected in this case due to the patient's preserved hemodynamic stability, gradual progression of symptoms, and the primary aim of confirming hemothorax progression rather than identifying an arterial bleeding source. At that stage, there were no clinical or radiologic features suggesting high-pressure arterial hemorrhage that would mandate immediate contrast-enhanced imaging or angiographic intervention. Nevertheless, contrast-enhanced CT remains the preferred modality in unstable patients or when arterial injury is suspected, and its role should be emphasized in similar clinical scenarios.

Published literature supports that venous or tumoral hemothorax can often be managed non-operatively when hemodynamic stability is maintained. Multidisciplinary collaboration among interventional radiology, thoracic surgery, and pulmonology teams ensures optimal patient outcomes [12].

## Conclusion

Massive hemothorax following CT-guided lung biopsy is an uncommon but clinically significant complication. In the absence of arterial bleeding, conservative management with drainage and vigilant observation can be safe and effective. This approach aligns with established trauma management principles and may prevent unnecessary surgical intervention in stable patients.

## CRediT authorship contribution statement

**Bahman Rasuli:** Writing – review & editing, Writing – original draft, Visualization, Validation, Supervision, Project administration, Conceptualization. **Ali Forat Yazdi:** Writing – review & editing.

## Patient consent statement

The patient provided written informed consent for the publication of this case report, including all clinical data and accompanying images.

## Declaration of competing interest

The authors declare that they have no known competing financial interests or personal relationships that could have appeared to influence the work reported in this paper.
